# Reducing Nurses' Emotional Exhaustion and Turnover Intentions: The Role of Prosocial Orientation and Perceived Patient Gratitude in a Moderated Mediation Model

**DOI:** 10.1155/jonm/4445460

**Published:** 2025-05-15

**Authors:** Ferdinando Toscano, Teresa Galanti, Michela Cortini

**Affiliations:** ^1^Department of Psychology, University of Campania “Luigi Vanvitelli”, Caserta, Italy; ^2^Department of Psychology, University of Chieti-Pescara “Gabriele d'Annunzio”, Chieti, Italy

**Keywords:** emotional exhaustion, patient gratitude, prosocial orientation, social exchange theory, turnover intention

## Abstract

**Aims:** This study investigates the relationship between prosocial orientation and turnover intention among Italian nurses, examining the mediating role of emotional exhaustion and the moderating effect of perceived patient gratitude.

**Design:** Cross-sectional quantitative study.

**Methods:** A sample of 162 nurses employed in Italian hospitals completed an online survey on the Qualtrics platform assessing prosocial orientation, patient gratitude, emotional exhaustion, and turnover intention. Data were analyzed through a moderated mediation model via the PROCESS macro in JASP.

**Results:** Prosocial orientation was negatively associated with turnover intention, with emotional exhaustion acting as a mediator in this relationship. Perceived patient gratitude moderated the impact of prosocial orientation on emotional exhaustion. The conditional indirect effect of prosocial orientation on turnover intention through emotional exhaustion was significant at average and high levels of patient gratitude, but not at low levels.

**Conclusion:** The findings suggest that fostering prosocial orientation perceptions and enhancing patient gratitude and its perceptions can mitigate emotional exhaustion and reduce turnover intentions among nurses.

**Implications for Nursing Management:** Healthcare organizations can promote prosocial orientation and patient gratitude through training, feedback, and recognition events to reduce nurses' emotional exhaustion and turnover.

## 1. Introduction

The global nursing shortage has reached critical levels, especially as the aging population grows. This is a worldwide issue, relentlessly compromising the quality of healthcare and universal health coverage [1]. In the U.S., the shortage is particularly severe, requiring 3 million more nurses and an estimated global deficit of 12.9 million nurses by 2035 [2]. Europe is also facing significant shortages, needing 590,000 nurses by 2020 [1]. Within Europe, Italy faces a particularly dangerous nursing shortfall, with a current deficit of 63,000 nurses and a nurse-to-population ratio of 6.3 per 1000 inhabitants, well below the EU average of 8.3 [3]. Additionally, Italy's nurse-to-doctor ratio of 3 nurses per doctor, along with below-average salaries, further strains the healthcare system [3].

Nursing shortage is a multifaceted issue driven by an aging workforce, inadequate training and recruitment, job dissatisfaction, economic factors, demographic changes, and negative perceptions of the profession [4–7]. Worryingly, a rise of one unit in nurse–patient workload is associated with a 3.4% increase in mortality risk per additional patient per nurse [8].

Training and bringing new nurses into the labor market is the main way to combat this shortage. Nonetheless, this professional category is often characterized by high levels of turnover [9], which undermines facilities losing their current experienced staff without being able to exhaustively control the number of nursing professionals they involve. Retaining experienced nurses is crucial for maintaining continuity of care, patient safety, and healthcare system efficiency. While efforts to train and recruit new nurses are important, retention strategies ensure that the expertise, critical thinking, and institutional knowledge of seasoned nurses are preserved [10, 11]. High turnover not only disrupts team dynamics but also increases workload for remaining staff, leading to burnout and further attrition [5]. Frequent turnover is costly for healthcare organizations due to the need for ongoing recruitment, onboarding, and training, making retention a more cost-effective strategy [7]. More importantly, nurse retention directly impacts patient outcomes, since, as a recent study shows, a 10% increase in nurses' intention to leave seems associated with a 14% increase in inpatient hospital mortality [8].

Healthcare systems must prioritize the retention of skilled professionals to ensure safe and high-quality care for patients. Among the various strategies available, one key approach is improving the professional recognition of the nursing profession. Enhancing the status and social standing of nurses not only has the potential to attract new entrants to the field but also can reduce turnover intentions among those already in service. Despite the growing importance of nurses in healthcare systems—driven by advancements in medical technology and increased life expectancy—the profession's societal recognition has lagged behind [12]. This lack of recognition undermines nurses' perception of their usefulness to others, weakening their professional identity and contributing to emotional exhaustion, which significantly increases turnover rates [13].

**Figure 1:**
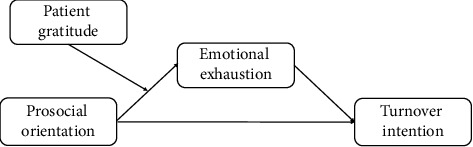
Research model.

In this study, grounded in social exchange theory (SET) [14, 15], we aim to assess whether prosocial orientation within the nursing workforce is associated with reduced emotional exhaustion and turnover intentions. Prosocial orientation reflects how much nurses perceive their actions as meaningful and beneficial to others' well-being. It is hypothesized to enhance nurses' resilience by fostering deeper, more meaningful relationships with patients, thereby alleviating emotional exhaustion. Furthermore, we explore the moderating role of perceived patient gratitude in strengthening the protective effect of prosocial orientation on emotional exhaustion. Specifically, we propose that higher levels of perceived patient gratitude may amplify the positive impact of prosocial orientation on reducing nurses' emotional exhaustion, which in turn diminishes turnover intentions. By exploring the moderated mediation model, depicted in Figure 1, this study seeks to identify actionable strategies to enhance nurse retention and aims to promote a more emotionally sustainable work environment. The findings are expected to provide insights into how interventions can be designed to foster both prosocial orientation and patient gratitude, optimizing psychological well-being and retention among nurses.

## 2. Background

High turnover intention among nurses poses a critical threat to healthcare systems worldwide, as it directly impacts the quality of patient care, disrupts team dynamics, and imposes significant costs on healthcare organizations due to ongoing recruitment and training [16–18]. Given the complex nature of caregiving roles, where nurses frequently engage in emotionally demanding interactions [19], understanding the antecedents of turnover intention is crucial for developing effective retention strategies.

One key factor that can influence turnover intention is prosocial orientation—a perception of one's actions as meaningful and beneficial to others' well-being. Nurses with a strong prosocial orientation are naturally inclined to invest emotional resources in their caregiving roles, deriving personal meaning and fulfillment from their work [20]. Previous research shows that nurses who provide patient-centered care often display lower turnover intentions as these nurses feel more connected to their workplace and are motivated to pursue organizational goals [21]. The alignment between their personal values and professional roles fosters a sense of commitment and dedication, which is generally associated with lower turnover intention [22]. However, the effectiveness of prosocial orientation in reducing turnover intention is not absolute and depends on how well nurses can manage the emotional demands of their work.

Based on SET [15], we claim that the relationship between nurse prosocial orientation and turnover intention can be explained through a mechanism of reduction of emotional exhaustion, so that it acts as a mediator of this relationship. Emotional exhaustion, defined as a state of feeling overextended and depleted of emotional resources [23], emerges when individuals are unable to recover adequately from the emotional strain associated with their work [24]. Based on SET [14, 15], we claim that, in the nursing profession, where the nature of caregiving involves significant emotional investments, emotional exhaustion can undermine the perceived value of the exchange between nurses and the healthcare facilities. When nurses feel that their emotional investments are not reciprocated—through recognition, support, or appreciation—the exchange becomes imbalanced, leading to increased emotional exhaustion [25]. This depletion of emotional resources, in turn, is a primary driver of turnover intention, as nurses who experience high levels of emotional exhaustion are more likely to consider leaving their job [26].

Previous research shows that a high prosocial orientation can reduce emotional exhaustion by enabling nurses to perceive their work as meaningful and aligned with their values, thus enhancing their resilience against the emotional demands of caregiving [27]. When nurses can maintain their emotional resources despite the demanding nature of their roles, they are less likely to experience exhaustion and more likely to remain in their positions.

However, also the effectiveness of prosocial orientation in reducing emotional exhaustion depends on contextual factors that influence the reciprocity of social exchanges. A study found that prosocial motivation significantly enhances emotional stability and reduces emotional exhaustion when coupled with a supportive work environment [28]. Nurses with high prosocial motivation were more likely to thrive in their roles, even in stressful environments, as long as they received adequate support and acknowledgment from their supervisors and colleagues [28]. On the other hand, also the ability to handle emotional demands is a critical factor, since also high emotional demands can lead to emotional exhaustion [25, 29].

Trying to advancing the factors according to which prosocial orientation is negatively related to emotional exhaustion, we adopt SET also for underlining that social exchanges are most effective when perceived as reciprocal and balanced, where the return on investment matches the initial contribution [15]. In the nursing context, this means that nurses' emotional investments should be met with adequate acknowledgment and appreciation not only by colleagues and other health facilities staff but also from patients. For this reason, we claim that perceived patient gratitude serves as a critical moderating variable that influences the relationship between prosocial orientation and emotional exhaustion.

Within SET, gratitude from patients acts as a form of positive reciprocation, signaling to nurses that their efforts and emotional labor are valued and appreciated [30, 31]. This perceived gratitude provides a tangible return for the nurses' emotional investments, restoring balance to the social exchange and fulfilling the nurses' need for acknowledgment. In other words, when patient gratitude is present, it helps to reinforce the positive aspects of the nurse–patient relationship and prevents emotional exhaustion by compensating for the emotional labor expended in caregiving [32–34]. Thus, perceived patient gratitude not only buffers against emotional exhaustion but also amplifies the protective effect of prosocial orientation on nurses' emotional well-being.

The moderating effect of perceived patient gratitude is then crucial for understanding the conditions under which prosocial orientation can translate into lower turnover intentions. We posit that when perceived patient gratitude is high, the positive impact of prosocial orientation on reducing emotional exhaustion is amplified, which, in turn, leads to decreased turnover intentions. Conversely, when gratitude is low or absent, the positive effect of prosocial orientation is diminished, and emotional exhaustion is more likely to increase, ultimately escalating turnover intentions. This suggests that the role of patient gratitude in maintaining balanced social exchanges is essential for sustaining the emotional resources of nurses and preventing turnover.

### 2.1. Aims

For the said reasons, in our study, we test a moderated mediation relationship, where perceived patient gratitude not only reduces emotional exhaustion directly but also conditionally amplifies the indirect effect of prosocial orientation on turnover intention through emotional exhaustion. Specifically, when perceived patient gratitude is high, the indirect effect of emotional exhaustion on turnover intention is weakened, and the beneficial effects of prosocial orientation are more pronounced. This highlights the importance of fostering patient gratitude, as well as recognizing it, within healthcare settings, as it can optimize the emotional well-being of nurses and reduce their intention to leave the profession [32, 34]. Furthermore, it underscores the importance of interventions that promote patient gratitude and reinforce prosocial orientation to support nurse retention and create a more emotionally sustainable work environment for nurses.

## 3. Method

### 3.1. Participants and Procedure

This cross-sectional study was conducted with a sample of 162 nurses employed in Italian public hospitals. Data collection was carried out using an online survey hosted on the Qualtrics platform through the snowball technique. To preserve the perception of anonymity among participants, no specific information about the hospitals where the respondents worked was recorded.

### 3.2. Ethical Considerations

This research was reviewed and approved by the Institutional Review Board of Psychology (IRBP) at the Department of Psychological, Health, and Territorial Sciences, University G. d'Annunzio of Chieti-Pescara (Protocol Number: 24,015; Approval Date: April 11, 2024). The study adheres to the guidelines of the Declaration of Helsinki. Participants provided written informed consent electronically via Qualtrics prior to completing the survey. The consent form outlined the study's purpose, voluntary nature, confidentiality assurances, and the right to withdraw at any time. Without providing this consent, the questionnaire automatically terminated, preventing participants from proceeding further.

### 3.3. Measures

The study utilized the following measures.

Prosocial orientation was measured using the four-item subscale from Dik et al. [35], such as “Making a difference for others is the primary motivation in my work.” Responses were rated on a 7-point Likert scale ranging from 1 (strongly disagree) to 7 (strongly agree).

Perceived patient gratitude was assessed with the four-item scale from Martini and Converso [36]; for example, “Several patients express gratitude for the care we provide.” Responses were recorded on a 7-point Likert scale ranging from 1 (strongly disagree) to 7 (strongly agree).

Emotional exhaustion was evaluated using the four-item scale from Gil-Monte and Figueiredo-Ferraz [37], such as “I feel emotionally exhausted.” Participants rated their responses on a 5-point scale ranging from 1 (never) to 5 (frequently).

Turnover intention was measured with the three-item scale from Cropanzano et al. [38]; for example, “I frequently think about leaving my job.” Responses were recorded on a 5-point scale ranging from 1 (never) to 5 (frequently).

### 3.4. Data Analysis

Before testing the research model, we addressed potential common method bias using Harman's single-factor test, which involved conducting an exploratory factor analysis (EFA) with the principal axis method. We then assessed the measurement model's validity and reliability by performing two confirmatory factor analyses (CFAs) and calculating Cronbach's alpha, composite reliability (CR), and average variance extracted (AVE) for each study scale. Descriptive statistics (mean and standard deviation) were computed to evaluate participants' demographic characteristics and perceptions, along with the correlations between variables. Finally, we tested our hypotheses using the PROCESS macro (Model 7, with variables involving moderation centered) in JASP 0.19.

## 4. Results

### 4.1. Validity and Reliability of the Scales

A single-factor EFA was conducted as part of the Harman test to evaluate the potential presence of common method bias in our dataset. The analysis revealed that the single extracted factor accounted for 35% of the total variance, which is below the commonly accepted threshold of 50% cited in prior research [39], suggesting minimal common method bias risk.

To further assess the structural independence of the four constructs in our model and rule out the presence of a common latent factor, we conducted two CFAs. We compared a 1-factor model, where all items were loaded onto a single factor, with a 4-factor model, where items were grouped under their respective constructs. As expected, the 1-factor model showed poor fit (*χ*^2^ = 797.50; df = 90; *χ*^2^/df = 8.87; CFI = 0.45; TLI = 0.36; RMSEA = 0.22; SRMR = 0.15), whereas the 4-factor model demonstrated a very good fit (*χ*^2^ = 166.61; df = 62; *χ*^2^/df = 2.69; CFI = 0.94; TLI = 0.92; RMSEA = 0.08; SRMR = 0.06; minimum item loading = 0.45), supporting the validity of our measurement model. Additionally, the reliability (Cronbach's alpha and CR) and validity (AVE) values, reported in Table 1, confirmed the robustness of our scales.

### 4.2. Description of Participants and Variables' Descriptive Statistics

The sample consisted of 162 valid respondents with an average age of 43.70 years (SD = 11.87), ranging from 22 to 65 years old. Most of the participants were female (78%). On average, participants had 14.78 years of tenure (SD = 11.76). Just under a quarter of the respondents (22.2%) reported living with children under 14 years of age. Most participants held a bachelor's degree, while 18 had a master's degree or higher qualifications. Lastly, 4.3% reported holding a coordinating or managerial role. Descriptive statistics revealed good levels of prosocial orientation and perceived patient gratitude, values above average of emotional exhaustion and relatively low levels of turnover intention.

The correlation matrix showed significant associations between the variables, with prosocial orientation positively correlated with perceived patient gratitude (*r* = 0.27, *p* < 0.001) and negatively correlated with emotional exhaustion (*r* = −0.24, *p* < 0.001) and turnover intention (*r* = −0.36, *p* < 0.001) (see Table 1).

### 4.3. Model Testing

**Figure 2:**
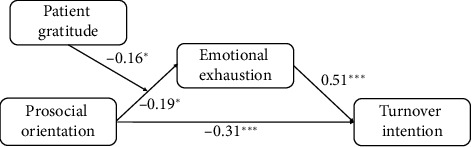
Research model: direct effects. Note: ^∗^*p* < 0.05; ^∗∗∗^*p* < 0.001.

**Figure 3:**
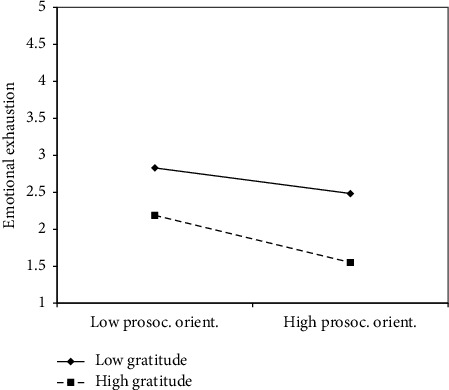
Moderating effects of patient gratitude on the relationship between prosocial orientation and emotional exhaustion.

To ascertain if the hypothesized relationships were supported by data, we computed the moderated mediation model using the PROCESS macro for JASP. The study results revealed that prosocial orientation was directly and negatively associated with turnover intention (*B* = −0.31, SE = 0.08, *p* < 0.001), indicating that individuals with a higher prosocial orientation were less likely to express intentions of leaving their job. Prosocial orientation was negatively related to emotional exhaustion (*B* = −0.19, SE = 0.08, *p*=0.01), and the latter was positively related to turnover intentions (*B* = 0.51, SE = 0.08, *p* < 0.001). Considering the moderating effect of patient gratitude, it was demonstrated, since it moderated the relationship between prosocial orientation and emotional exhaustion (*B* = −0.16, SE = 0.07, *p*=0.02). This suggests that when nurses perceived high levels of gratitude from patients, the protective effect of prosocial orientation on emotional exhaustion was strengthened.  Figure 2 depicts the research model with results, while Figure 3 shows the moderating effects.

We further examined the conditional indirect effects of prosocial orientation on turnover intentions through emotional exhaustion at different levels of perceived patient gratitude (16th, 50th, and 84th percentiles). At the average level of patient gratitude, the indirect effect of prosocial orientation on turnover intentions was negative and significant (Estimate = −0.12, SE = 0.05, *p* < 0.01), indicating that individuals with higher prosocial orientation experienced lower levels of emotional exhaustion, which subsequently reduced turnover intentions. However, at the 16th percentile of patient gratitude, the conditional indirect effect was not statistically significant, suggesting that low levels of patient gratitude may not provide sufficient support to leverage the positive impact of prosocial orientation in reducing emotional exhaustion and turnover intentions. Conversely, at the 84th percentile, the indirect effect was stronger and significant (Estimate = −0.16, SE = 0.06, *p* < 0.01), indicating that high levels of perceived patient gratitude further amplified the protective effect of prosocial orientation on emotional exhaustion, leading to a greater reduction in turnover intentions.

These findings are reflected in the conditional total effects of prosocial orientation on turnover intentions, which ranged from Estimate = −0.35 (SE = 0.09, *p* < 0.001) at the 16th percentile of patient gratitude to Estimate = −0.47 (SE = 0.09, *p* < 0.001) at the 84th percentile. Conditional indirect and total effects are reported in Table 2.

## 5. Discussion

Based on SET and in a sample of 162 nurses working in Italian public hospitals, this study found that prosocial orientation was negatively associated with turnover intentions, and this relationship was mediated by emotional exhaustion. The effect was further moderated by patient gratitude, with higher levels of perceived gratitude strengthening the protective impact of prosocial orientation on reducing emotional exhaustion and, consequently, turnover intentions.

The findings of this study provide important insights into the role of prosocial orientation and patient gratitude in mitigating emotional exhaustion and reducing turnover intention among Italian public hospital nurses. Our results reveal that prosocial orientation may directly reduce turnover intention by lessening emotional exhaustion, and that this relationship can be further influenced by the moderating role of patient gratitude. These findings underscore the value of promoting both prosocial orientation and patient gratitude to create a more emotionally sustainable work environment for nurses, ultimately supporting nurse retention.

The negative relationship between prosocial orientation and emotional exhaustion is consistent with SET and previous research suggesting that a strong prosocial orientation helps individuals find deeper meaning in their work [40]. Nurses with high prosocial orientation may perceive their work as aligned with their personal values and fulfilling, which can enhance their resilience to emotional demands [41]. This aligns with our hypothesis that prosocially oriented nurses are less prone to emotional exhaustion. The mechanism appears to be that when nurses see their work as intrinsically rewarding and beneficial to others, they are better equipped to cope with the high emotional demands of the profession.

However, this buffering effect of prosocial orientation on emotional exhaustion is not unconditional. Our findings show that perceived patient gratitude is a key moderating variable that amplifies the positive impact of prosocial orientation. This suggests that while prosocial orientation is crucial for reducing emotional exhaustion, the extent to which it is effective depends on the contextual factor of patient gratitude which, in the light of SET, serves as a form of reciprocation that reinforces nurses' perception of their work as valuable.

Our study found that the protective effect of prosocial orientation on emotional exhaustion is significantly stronger when perceived patient gratitude is high. This highlights the importance of reciprocal social exchanges in reducing emotional exhaustion [14, 42]. Patient gratitude can be seen as an acknowledgment of nurses' emotional labor, providing tangible evidence that their efforts are valued. When patient gratitude is high, it restores balance in the nurse–patient social exchange, compensating for the emotional resources expended and enhancing the perceived value of the exchange. Conversely, at low levels of patient gratitude, the indirect effect of prosocial orientation on emotional exhaustion is not significant. This indicates that when nurses perceive a lack of gratitude from patients, their emotional resources are more likely to become depleted, leading to increased emotional exhaustion and higher turnover intentions.

Moreover, this buffering effect may depend on how nurses perceive and process gratitude. Some may overlook subtle expressions of gratitude, while others may draw strength from small gestures. Therefore, gratitude awareness may add an additional layer to the social exchange process. The benefits of social exchanges are not solely about external reciprocation but also about how these gestures are cognitively recognized. Perceived gratitude may play a key role as a cognitive-emotional filter, suggesting that nurses who are more aware or sensitive to patient gratitude may be better able to convert these positive interactions into emotional resources. This aligns with cognitive appraisal theory [43], which posits that the way individuals interpret their environment influences their emotional responses. Nurses who perceive patient gratitude as meaningful may manage emotional exhaustion more effectively, reinforcing prosocial behavior as a rewarding experience [44].

These findings suggest that healthcare organizations should promote interventions that focus both on fostering patient gratitude and enhancing nurses' awareness of the gratitude they receive. For instance, patient feedback programs or recognition initiatives could encourage patients to express appreciation for the care provided by nurses. At the same time, organizations could implement mindfulness practices or awareness training to help nurses become more attuned to the gratitude they receive, ensuring that these positive interactions are recognized and internalized.

### 5.1. Theoretical Implications

This study introduces a new perspective on reducing turnover intentions among nurses by emphasizing the role of prosocial orientation and patient gratitude. Traditionally, research on nurse well-being and turnover has focused on organizational and job-specific factors [45–47]. Our findings place prosocial orientation at the center of these outcomes, suggesting a renewed emphasis on theories related to work identity and intrinsic motivation [48].

Furthermore, this study shifts the application of SET by incorporating perceived patient gratitude as a key factor in nurse well-being. While SET has typically been used to explain the dynamics between employees and employers, our research demonstrates that positive exchanges with patients, expressed through their gratitude, can significantly influence nurses' emotional states and commitment to their roles. This reframing action positions patient gratitude as a crucial relational resource, shaping the emotional and professional experiences of nurses.

By expanding the scope of SET to include patient–nurse interactions, this study highlights the importance of recognizing gratitude as a form of nonmaterial reciprocity that reinforces the prosocial orientation of healthcare workers. It suggests that patient gratitude should be considered a central component in the social exchange process, capable of reducing emotional exhaustion and turnover intentions. Furthermore, introducing nurses' perception of gratitude adds nuance to SET by suggesting that the benefits of social exchanges are not just about external reciprocation (e.g., gratitude shown by patients), but also related to how these gestures are cognitively recognized. This shifts part of the responsibility for emotional well-being onto the individual cognitive framework of nurses. This perspective encourages future research to explore other forms of patient feedback and appreciation as potential moderators in the relationship between prosocial behaviors and employee retention in healthcare settings.

### 5.2. Practical Implications

The results of this study have significant implications for nursing retention strategies. While previous research has largely focused on organizational factors such as job satisfaction and workload management, our findings highlight the critical role of individual and interpersonal elements, including prosocial orientation and patient gratitude. By enhancing prosocial orientation through targeted training programs, such as workshops on empathy and compassionate communication, and promoting patient gratitude through educational initiatives and active patient engagement, healthcare organizations can foster a more supportive and emotionally sustainable work environment for nurses.

Our findings also suggest that promoting prosocial orientation alone may not be sufficient to alleviate emotional exhaustion unless complemented by positive reciprocation from patients. This underscores the need for a comprehensive approach that includes both individual-level interventions to strengthen prosocial orientation and contextual interventions to elevate patient gratitude. Healthcare organizations could implement strategies such as structured feedback systems, recognition events, and opportunities for patients to express their gratitude, which can enhance the nurse–patient relationship and contribute to nurses' emotional well-being. At the same time, organizations could implement training programs designed to help nurses recognize and internalize gratitude. These programs might include mindfulness practices or reflective exercises aimed at enhancing nurses' awareness of the positive feedback they receive. For example, structured time for reflection, such as post-shift debriefs or diary exercises [49], where nurses reflect on patient interactions and moments of gratitude, could foster a greater sense of meaning in their work. Such an integrated strategy can optimize psychological health, thereby reducing turnover intention and mitigating the adverse effects of nursing shortages on healthcare systems.

Considering these observations, it is evident that personal orientation and intrinsic motivation remain central to nurses' commitment to their profession. A strong alignment between personal values and professional identity can be a powerful tool in preventing burnout and improving retention. Professional bodies, such as nursing boards and professional associations, can play a key role in reinforcing this alignment by promoting the core values of nursing, organizing initiatives to strengthen professional identity, and reminding nurses of the reasons behind their career choice.

Moreover, enhancing patient gratitude through systematic feedback mechanisms and recognition events is crucial. Events such as “Patient Appreciation Days” or campaigns that encourage patients to share their positive experiences can contribute to a culture of appreciation. At the same time, introducing digital tools (e.g., QR codes in patient rooms or bedside tablets) can enable patients and their families to express gratitude instantly rather than sporadically. These gratitude messages could then be prominently displayed on digital “Gratitude Boards” in staff areas or featured regularly in organizational newsletters, reinforcing nurses' awareness of their meaningful impact. More broadly, fostering a general “spirituality” of work emphasizing purpose, meaningfulness, and interconnectedness can significantly enhance nurses' engagement and emotional fulfillment.

However, it is important to strike a balance between emphasizing nurses' social contributions and avoiding the perception of nursing as a purely altruistic vocation. This distinction is crucial to maintaining the professional integrity of the role and ensuring that recognition efforts do not devolve into superficial narratives that undermine the complexity and professionalism of nursing. Accordingly, balancing the emphasis on nursing values with avoiding a patronizing view of nurses as “selfless caregivers” instead of skilled professionals requires targeted strategies and concrete resources. This means investing in professional development, creating clear career advancement paths, and ensuring that recognition is based on professional competencies rather than a sentimentalized view of work. In sum, a balanced approach that respects both personal values and professional skills can support nursing staff and create sustainable and fulfilling environments.

### 5.3. Study Limitations

Despite the strengths of this study, several limitations should be acknowledged. First, the cross-sectional design limits our ability to draw causal inferences. We acknowledge this limitation and suggest that future studies could employ longitudinal designs to provide stronger causal evidence regarding how prosocial orientation and patient gratitude evolve over time and influence turnover intentions. Additionally, future research could explore how individual factors such as personality traits, emotional intelligence, or mindfulness affect nurses' ability to recognize and internalize patient gratitude. Second, our findings are potentially confined to the public healthcare sector in Italy, which could limit the applicability of the results to other settings, such as private healthcare or other national contexts. Cultural and systemic differences may affect the dynamics observed in this study. Therefore, future research should explore whether these relationships hold in different sectors and countries to enhance the generalizability of the findings.

Furthermore, our study focused on patient gratitude as the sole moderating factor. Other contextual variables, such as interactions with other healthcare providers, coordinators, and organizational or ward dynamics, could also influence the relationship between prosocial orientation and emotional exhaustion. Future research could examine these variables to provide a more comprehensive understanding of the factors that affect nurse retention. Finally, the reliance on self-reported measures may have introduced common method bias. We acknowledge this limitation, and we specify that we not only conducted Harman's single-factor test to mitigate concerns about common method variance but also employed different response formats and scales to minimize pattern recognition by respondents and strategically placed scales in different parts of the survey to prevent response bias due to item positioning. Future studies should continue to address potential biases by incorporating objective or multisource data where feasible.

## 6. Conclusion

This study demonstrates that fostering prosocial orientation and perceived patient gratitude can effectively mitigate emotional exhaustion and reduce turnover intentions among nurses. Healthcare organizations should prioritize strategies that promote these factors to create a more supportive and emotionally sustainable work environment for nurses. By doing so, they can enhance nurse retention and ensure high-quality patient care in the face of growing nursing shortages.

## Figures and Tables

**Table 1:** Reliability indices, descriptive statistics, and correlations among variables.

	M (SD)	AVE	CR	1	2	3	4
1. Prosocial orientation	5.67 (0.88)	0.41	0.71	*(0.69)*			
2. Perc. patient gratitude	5.67 (1.05)	0.72	0.91	0.27^∗∗∗^	*(0.91)*		
3. Emotional exhaustion	3.23 (0.91)	0.67	0.89	−0.24^∗∗^	−0.32^∗∗∗^	*(0.88)*	
4. Turnover intention	2.23 (1.06)	0.62	0.82	−0.36^∗∗∗^	−0.22^∗∗^	0.50^∗∗∗^	*(0.82)*

*Note*: Cronbach' s alphas in brackets on the diagonal. Cronbach's alpha (in italics, in brackets) indicate nonsignificant values (in statistics).

^∗∗^ *p* < 0.01.

^∗∗∗^ *p* < 0.001.

**Table 2:** Conditional indirect and total effects at the 16^th^, 50^th^, and 84^th^ percentile.

Patient gratitude value	Cond. indirect effect	Cond. total effect
Low patient gratitude (16^th^ percentile)	−0.04	−0.35^∗∗∗^
Average patient gratitude (50^th^ percentile)	−0.12^∗∗^	−0.43^∗∗∗^
High patient gratitude (84^th^ percentile)	−0.16^∗∗^	−0.47^∗∗∗^

^∗∗^ *p* < 0.01.

^∗∗∗^ *p* < 0.001.

## Data Availability

The data that support the findings of this study are available from the corresponding author upon reasonable request.
